# Java GUI for InterProScan (JIPS): A tool to help process multiple InterProScans and perform ortholog analysis

**DOI:** 10.1186/1471-2105-7-462

**Published:** 2006-10-20

**Authors:** Aijazuddin Syed, Chris Upton

**Affiliations:** 1 Department of Biochemistry and Microbiology, University of Victoria, Victoria, BC, V8W 3P6, Canada

## Abstract

**Background:**

Recent, rapid growth in the quantity of available genomic data has generated many protein sequences that are not yet biochemically classified. Thus, the prediction of biochemical function based on structural motifs is an important task in post-genomic analysis. The InterPro databases are a major resource for protein function information. For optimal results, these databases should be searched at regular intervals, since they are frequently updated.

**Results:**

We describe here a new program JIPS (Java GUI for InterProScan), a tool for tracking and viewing results obtained from repeated InterProScan searches. JIPS stores matches (in a local database) obtained from InterProScan searches performed with multiple versions of the InterPro database and highlights hits that have been added since the last search of the InterPro database. Results are displayed in an easy-to-use tabular format. JIPS also contains tools to assist with ortholog-based comparative studies of protein signatures.

**Conclusion:**

JIPS is an efficient tool for performing repeated InterProScans on large batches of protein sequences, tracking and viewing search results, and mining the collected data.

## Background

Recent advances in DNA sequencing technology have led to an unprecedented and rapid accumulation of genomic data [1-3]. Although this huge amount of data is immensely useful for a variety of comparative *-omics *studies, it also presents significant challenges in the areas of data management and analysis, as databases need to be designed to accommodate future growth. Comparative analysis tools must also be able to handle increasing amounts of data; the processing power of computers may be increasing, but such analyses are often computationally intensive. Another aspect of using these tools that is sometimes forgotten is that analyses such as BLAST similarity searches [4] or InterPro motif scans [5-8] are not *one-shot *experiments. Since the sequence/motif databases they use are continually changing, results quickly become obsolete and thus searches must be repeated at frequent intervals.

The manual running of such analyses on a regular basis may not present a problem to a researcher who is only interested in a few specific genes. However, larger-scale query sets (e.g. an entire gene family), may contain so many sequences that the process becomes a highly tedious chore. One serious consequence of this is that such analyses are often performed only sporadically, and thus significant new database matches are not discovered in a timely fashion. We designed the program Recent Hits Acquired from BLAST (ReHAB) [9] to automate PSI-BLAST [4] searches and help mine the results. ReHAB has the following features: 1) it automatically performs regular PSI-BLAST searches on large numbers of query proteins; 2) it allows the user to browse the search results, via a simple interface; 3) it highlights new database hits, distinguishing them from the large volume of unimportant PSI-BLAST output; and 4) it assists with further investigation of the results (comparing orthologs and creating multiple sequence alignments (MSA) for selected hits.)

Along with similarity searches such as BLAST and FASTA [10], one of the most useful methods of predicting protein function is examining a sequence for the presence of signature motifs. Most genomics researchers are probably familiar with the PROSITE [11] and Pfam [12] databases. InterPro [5-7] is a searchable *super-database *that integrates a variety of signature-based databases and can be queried using a sequence via the InterProScan tool. Since the InterPro database is subject to regular updates because new motifs are discovered and old ones refined, past searches should be repeated with each database release. Searches should be performed using all available members of a particular protein family, as this increases the overall chance of matching a database protein signature. InterProScan can be operated via a web interface [13] and although a locally installed version can run large numbers of proteins in batch mode, the reviewing of results can be extremely tedious and time-consuming. In addition, the results must be viewed individually or parsed by a separate computer program.

These considerations prompted us to design a new program, Java GUI for InterProScan (JIPS), to aid in the analysis of protein sequences by InterProScan and thus alleviate these problems. Specifications for the software included: 1) an interface to simplify batch runs and analyses; 2) a mechanism to flag new signature matches for the user; 3) tools to assist in ortholog comparisons and further analysis; 4) the ability to export signatures as annotations to the query protein. JIPS stores the query sequences together with the results produced by searching the InterPro database in local *JIPS databases*.

## Implementation

### Rationale

JIPS was implemented using Java to support multiple operating systems (including Mac OS X, Linux, Solaris and Microsoft Windows), and to ensure compatibility with other Java-based Viral Bioinformatics Resource Center [14] applications, including the Virus Orthologous Clusters database (VOCs) [14,15], and Base-By-Base (BBB) [14,16]. Users initially access and launch the application (JIPS client) from a web page using Java Web Start (JWS). A local application is then created on the user's computer; updated versions of the software are automatically downloaded as they become available.

### JIPS Architecture

**Figure 1:**
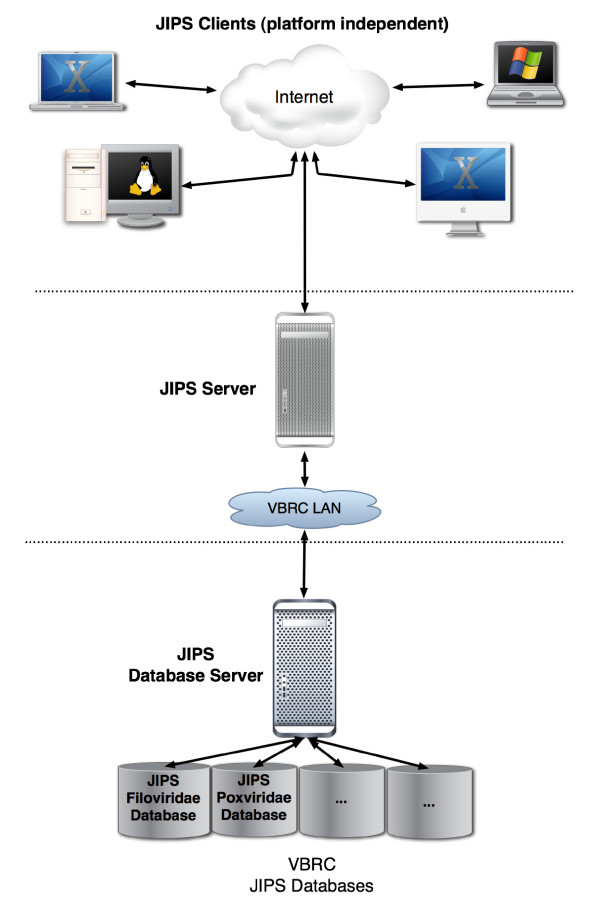
JIPS Architecture Diagram.

JIPS (Figure 1) was designed with a three-tier client/server architecture [17] modeled on ReHAB [9]. The three primary components of JIPS are: 1) the JIPS client (the front-end); 2) the JIPS server that accepts requests from the client and manages system processes; and 3) the JIPS database server that stores the results of InterProScan runs and query protein information. Although all of the components can be located on a single machine, a more common arrangement is that used at the VBRC in which a single JIPS server and database are used to service a variety of JIPS clients via our Intranet and the Internet. If greater capacity is required, it is a straightforward task to distribute InterProScan jobs onto a Grid/Cluster system with relatively minor changes to the program.

Currently, since the InterPro database is updated on approximately a 3 month cycle, the downloading of new versions is performed manually. Similarly, each run of the proteins in a JIPS database against a new InterPro database is initiated using the administrator version of the JIPS client. This process takes only a few mouse clicks and allows for confirmation that the previous run, which may take several days depending on the number of proteins, has completed correctly.

### JIPS Client

The JIPS client is a Java Swing-based GUI that provides the user with an intuitive interface to browse InterProScan results, and allows managers to update the JIPS databases as required (the user must log in as an *administrator *to perform these functions; see below). The client contains five main components, arranged as follows (in the VBRC implementation): 1) the *JIPS management console *that lists all available local JIPS databases, and has options for creating/deleting databases or adding/editing biological sequence data from FASTA-formatted files or from the VBRC VOCs database to existing JIPS databases; 2) the *JIPS Virus/Organism Browser *window that displays all the organisms in a selected JIPS database and allows users to set viewing options; 3) the *Summary of InterPro Hits *window that displays the list of query genes from the selected organism, highlighting those genes which have new InterProScan hits; 4) the JIPS *Hits Manager *window, which displays detailed information about the hits for selected genes; and 5) the JIPS *Orthologs Comparison *window that allows users to compare the signature matches for protein orthologs.

**Figure 2:**
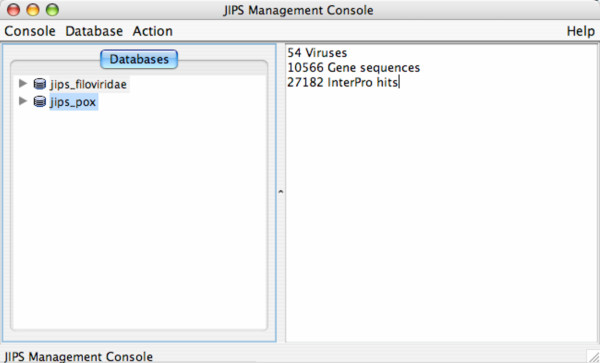
*JIPS Management Console *window. When a database is selected from the list on the left, a summary of the database is displayed in the right-hand pane. Double clicking the database or selecting *Browse Virus *from the *Action *menu will allow user to browse through the list of genes for the organism/virus.

JIPS supports two types of users: a privileged user who may use all functions of the program (administrator) and a general user. An administrator is permitted to: 1) create and delete JIPS databases; 2) update or import data into JIPS databases; and 3) start new InterProScan searches for the proteins in a JIPS database. A general user is permitted only to view and analyze existing InterProScan results. When JIPS is started from JWS, the *JIPS Console *window appears (Figure 2) together with a user authentication dialog box that allows administrators to log in; general users simply hit "Cancel" to close the login window. The *database pane *(Figure 2; left side) in the *JIPS Management Console *lists all of the available JIPS databases, and the *statistics pane *(Figure 2; right side) displays statistics (number of organisms/genes/hits) for the selected database.

Each database node in the *database pane *can be expanded by clicking on the adjacent arrow to show two child nodes. The *jobs node *is used by the administrator to initiate a new InterProScan search for each of the proteins in the selected JIPS database, and to check the status of running jobs. The *query sequences *node lists the proteins in the selected database. An administrator can group these sequences into gene families by selecting the genes and choosing "*Add Family ID to Selected Sequences*" from the Action menu; the Action menu changes according to items selected in the *database pane*.

Although we have focused on using the VOCs database as the source of protein sequences for the JIPS databases, the client (running administrator privileges) can be used to load large numbers of sequences (fasta multi-sequence format) into JIPS. The process is menu driven and allows the administrator to select the name to be associated with the protein set.

### JIPS Server

The JIPS server accepts requests from the JIPS client over a specific communications port. Requests are classified into two categories: data and computation request messages. After receiving a data request message, the server retrieves the requested data from the JIPS database server and returns it to the client. Computation request messages are used to manage server-side jobs (InterProScan searches) that require significant time to execute. Software requirements for the JIPS server are Java 1.4 and MySQL 4.0, with locally installed InterProScan software containing all InterPro databases. Detailed information is available on installing the JIPS server locally [18]; this is required if users wish to enter their own protein sequences into the JIPS system.

### JIPS Database Server

The JIPS database server is a MySQL relational database server. Within the VBRC implementation of JIPS, we have created separate databases for a series of taxonomic virus families (e.g. *Poxviridae*), each containing protein sequences from all fully-sequenced viruses belonging to the family. An alternative arrangement of sequence categories could just as easily be *Principal Investigator, Graduate Student, Favorite Proteins*, but neither categorization is necessary. JIPS is also capable of storing data about relationships between query proteins in its database. This ortholog information can either be obtained automatically (i.e. when viral proteomes are imported into JIPS from our VOCs database) or can be entered manually by an administrator (discussed below). JIPS is able to use these similarities to quickly sort the results (e.g. returning all available hits for *poxvirus DNA ligases*.)

Each of the JIPS databases store five types of information (see table 1) in five different database tables. All the InterProScan hits are stored in "*InterProScan hits*" table; the java SAX parser is used to read the XML InterProScan output. Since query sequences that belong to same gene family are likely hitting the same InterPro ids, additional information about the InterPro hit and corresponding signature hit (name and type) are stored separately in different tables to minimize data redundancy.

**Table 1:** Types of information stored in each JIPS database within the JIPS database server. Signatures that do not have an InterPro id are also stored

**Information type**	**Data stored in table**
Virus genome	Name, genome id, and GenBank accession number
Gene	Name, gene id, protein sequence, and gene family information
InterProScan hits	InterPro id, signature id, and date when hit was first recorded
InterPro entry	InterPro id, name, and type
Signature entry	Signature database id, name and type

## JIPS results and discussion

JIPS was conceived as a tool to help biologists manage and analyze the results generated by large numbers of InterProScan searches. It takes considerable hands-on time for a researcher to evaluate the results of even one InterProScan search when more than a few signatures are hit, and this problem is compounded when multiple proteins are searched. Reviewing results of repeated scans performed with different versions of InterPro is similarly tedious and time-consuming. Therefore, a primary goal in creating JIPS was to provide users with a *tracking tool *to quickly summarize differences between repeated searches. A second objective was to assist researchers in analyzing these results through comparative genomics.

JIPS is particularly useful for performing a series of InterProScan searches with a group of diverse protein orthologs. Investigating an InterPro signature that only appears in one, or a few, of the orthologs can be very productive. In some cases, comparison of the sequence containing the motif to the other orthologs may lead to the researcher detecting a variation of the signature pattern in these sequences. This would suggest that the original hit is significant and that the signature pattern may need to be generalized to reflect this new set of proteins. On the other hand, the researcher may conclude that the signature is indeed only present in the single sequence, suggesting that it is spurious (i.e. a random match). In both situations, running InterProScan on only a single member of the group would yield different, and possibly misleading, results.

### Browsing JIPS Hits

**Figure 3:**
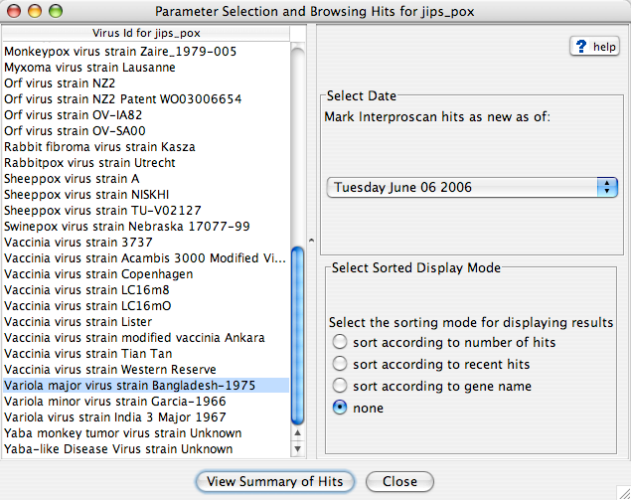
The *JIPS Virus/Organism Browser *window. Here the user can set viewing options for the result lists.

After starting JIPS, a general user can begin browsing a given JIPS database by either double-clicking on the database node in the left-hand panel of the *JIPS Management Console*, or selecting the *Browse virus/organism *item from the Action menu (Figure 2). The *JIPS Browse Organism/Virus *window then opens (Figure 3), providing the user with a list of all the viruses/organisms in this particular JIPS database. As an example, we chose the VBRC poxvirus database, containing 10,566 protein sequences from 54 complete poxvirus genomes. These were searched against the InterPro database with InterProScan, generating 27,182 signature hits. The next step is to select a virus/organism of interest and set display preferences for the InterProScan results. The user chooses 1) a *cutoff date*, used by JIPS to determine whether a given hit should be marked as *new*, and 2) the sorting mode (used to sort the list of genes that appears in the next window).

**Figure 4:**
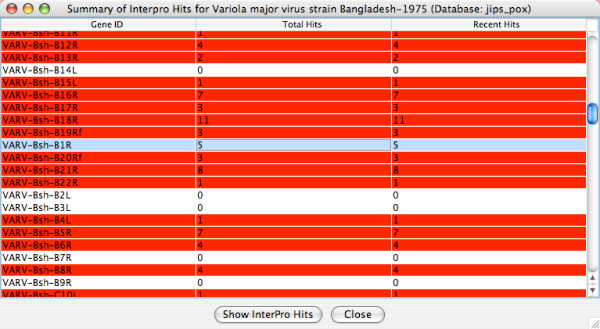
The *Summary of InterPro Hits *window for Variola major virus strain Bangladesh-1975. A scrollbar is used to view the remaining data.

**Figure 5:**
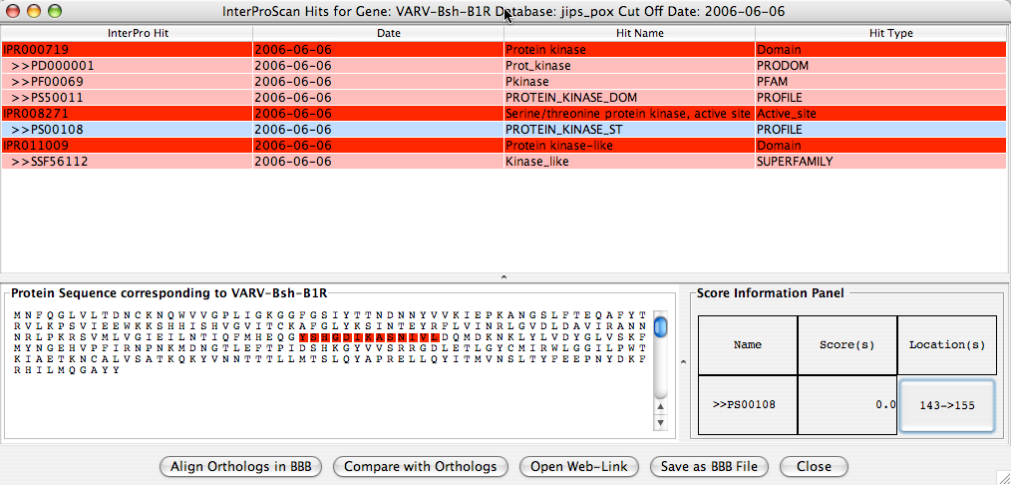
*JIPS Hits Manager *window for Variola major virus strain Bangladesh-1975 B1R; results show that the gene has 5 signature database hits, in three InterPro categories. The highlighted region of the query sequence below the table corresponds to PS00108.

Double-clicking on the virus/organism of interest, or clicking on the *View Summary of Hits *button, will open the *Summary of InterPro Hits *window for that virus/organism. Figure 4 shows a sample *summary of hits *for *Variola major virus strain Bangladesh-1975*. Each row in the window represents a protein encoded by this viral genome; from left to right, it lists the protein name, the total number of non-redundant hits, and the number of *new hits *(i.e. hits found since the cutoff date). A row is colored red if at least one of its hits was recorded after the cutoff date; otherwise, it is colored white. We selected the *VARV-Bsh-B1R *gene, which had a total of 5 signature hits, and clicked the *Show InterPro Hits *button. The *JIPS Hits Manager *window (Figure 5) opens, showing 5 individual signatures (pink) belonging to a total of 3 InterPro categories (red). If a given row in the table (corresponding to an InterPro category or individual hit) is selected, its hit score(s) and match region(s) are displayed, together with the query protein sequence, in the lower half of the window. Clicking on the *Location *button will highlight the region on the query sequence matching the signature hit. Finally, double-clicking on a row (or clicking the "Open Web Link" button) will open a web browser containing the signature home page; e.g. a Pfam signature from the Pfam database.

### Comparing/Aligning Orthologs

**Figure 6:**
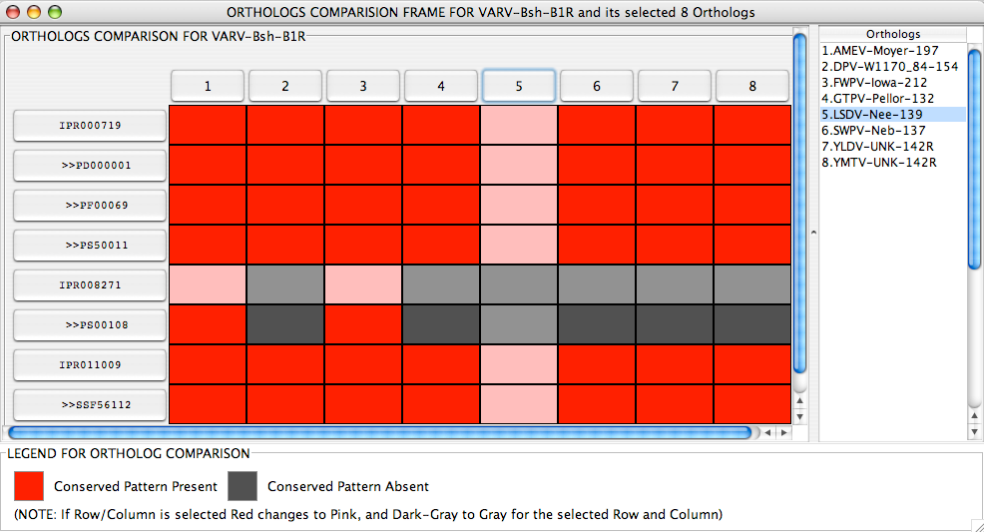
Matrix comparing Variola major virus strain Bangladesh-1975 B1R (variola virus protein kinase) signature hits to the results from a series of orthologous poxvirus proteins. This window is automatically sized to accommodate much larger numbers of protein sequences and InterPro hits. Cells in selected rows and columns are shown in paler shades.

At the bottom of the *JIPS Hits Manager *window are a series of buttons enabling the user to perform further analysis. Clicking on the *Compare with Orthologs *button opens a menu from which the user selects the database proteins to use in the comparison. A color-coded matrix-style graphic (*Orthologs Comparison *window) is then automatically generated, clearly showing which signatures are also present in the selected orthologs (Figure 6). In this example, we compared the InterProScan results for *VARV-Bsh-B1R *with those for orthologous proteins in the JIPS database. From the results in Figure 6, it is apparent that the PROSITE signature PS00108 (ser/thr protein kinase active site) is present in *VARV-Bsh-B1R *but absent from a number of the orthologs. Signatures present in an ortholog but missing from the original sequence can be easily found by clicking the button representing the protein of interest in the *Orthologs Comparison *window; a *JIPS Hits Manager *window for the ortholog will open.

**Figure 7:**
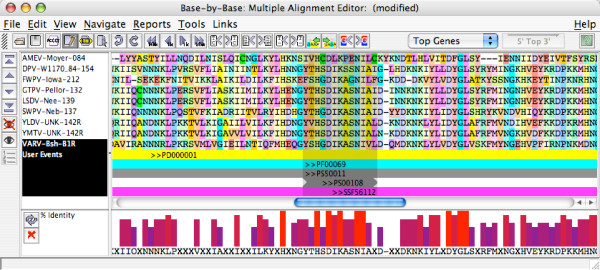
Alignment of the Variola major virus strain Bangladesh-1975 B1R protein kinase with eight orthologs, displayed in the Base-By-Base MSA editor. Signature hits and the consensus sequence are shown below the amino acid alignment.

Further analysis can be performed by clicking the *Align Orthologs in BBB *button (in the JIPS Hits Manager window); this will bring up a list of all available orthologs. JIPS will automatically retrieve the selected protein sequences from the database, submit them to MUSCLE [19] for alignment, and open the results in the MSA editor, BBB [14,16]. The signatures are shown as comments beneath the alignment and a consensus sequence can be displayed (Figure 7). From these results it is apparent that the reason that the PS00108 signature (regular expression: [LIVMFYC]-x- [HY]-x-D- [LIVMFY]-K-x(2)-N- [LIVMFYCT](3)) does not appear in some of the orthologs is due to a single amino acid substitution; they contain alanine as the penultimate residue of the motif.

### Saving Results

Although the JIPS database is itself a repository of InterPro search results, JIPS also provides a function (*Save as BBB File *button) that adds the signatures as *comments *(annotations) to a protein sequence and writes a file in BBB format. This allows simple reviewing and sharing of final results. The files are saved on the user's local computer and can be independently loaded into BBB. If required, the user can edit/add/delete these *comments *from within BBB and the other comment-associated features of this program are also available to the user.

The signatures can also be written to a BBB file as part of the MSA generated by the *Align Orthologs in BBB *feature (Figure 7). In this case, to conserve space in the viewer, the *comments *are only written for the primary protein

## Conclusion

InterPro is an extremely valuable and complex resource that integrates a wide variety of protein signature databases. JIPS was designed to mine the information in a comparative fashion from multiple InterProScan searches, thereby relieving the biologist of a variety of tedious information management jobs. To this end, JIPS is a powerful but simple-to-use tool that helps bioinformaticians and biologists navigate and analyze the volumes of data with which they are faced following medium, which may be a single family of orthologs, and large scale InterProScan searches. JIPS goes beyond data management and highlights new signatures matches for the user. It also integrates a series of tools to allow comparison of InterProScan searches for multiple proteins.

Through the viral databases maintained by Viral Bioinformatics – Canada [20], JIPS will support a large community of virologists, however, local installations will make it useful for a much wider audience.

## Availability and requirements

**Project name: **JIPS

Project home page: (workbench menu)

**Operating systems: **All platforms supporting Sun's JRE version 1.4.1 or compatible

**Programming languages: **Java, SQL

**Other requirements: **Java 1.4 or higher

**License: **Open Software License ()

## Authors' contributions

CU specified the features of and problems to be solved by JIPS, tested the program and provided usage examples. AS implemented the software, including both the Java components and the database schema used to store results. Both authors contributed to writing of the manuscript.
